# Positive mental health in adults with bipolar disorder: exploring social support subtypes, negative social interactions and potential to flourish

**DOI:** 10.1186/s12888-023-05244-3

**Published:** 2023-10-17

**Authors:** Ken Fowler, Kaya E. Dooley

**Affiliations:** 1 Student Wellness and Counselling Centre, Memorial University of Newfoundland, St. John’s, NL Canada; 2 Department of Psychology, Faculty of Science, Memorial University of Newfoundland, St. John’s, NL Canada

**Keywords:** Bipolar disorder, Social Support, Positive Mental Health, Negative social interactions, Gender differences

## Abstract

**Background:**

Bipolar disorder (BD) (i.e., BD-I or BD-II) is a serious mental illness (SMI) that can cause significant life challenges, but its impact and management may be mediated by psychosocial factors. This study’s primary objectives were to investigate whether adults with BD differ from those without in terms of social support, negative social interactions (NSIs), and positive mental health (PMH). Secondly, examine whether gender differences exist in terms of these variables, as well as whether specific social support subscales and NSI predict PMH for those with BD.

**Methods:**

Using data extracted from a national Canadian survey, 563 adults reporting a lifetime BD diagnosis were compared to a matched, non-BD sample using the Social Provisions Scale 10 Items (SPS-10), the NSI Scale, and the Mental Health Continuum – Short Form (MHC-SF) Scale. For the BD sample, males and females were compared based on study variables, and hierarchical regressions were subsequently performed to assess whether SPS-10 subscales and NSIs predicted PMH.

**Results:**

Respondents with BD reported significantly lower SPS-10 and PMH scores, and significantly higher NSI scores. Within the BD sample, females reported significantly higher SPS-10 and NSIs scores, and ‘social integration’ and ‘reassurance of worth’ positively predicted PMH, while NSI uniquely predicted lower PMH levels for both males and females.

**Conclusions:**

The results implicate specific psychosocial factors and gender in the degree to which adults with BD might flourish, particularly in terms negative relationships. The implications of social erosion and the bi-directionality of social support are also considered.

## Introduction

Bipolar disorder (BD) is a serious mental illness (SMI) that involves recurrent episodes of mania or hypomania and depression, and may be characterized by risk-taking behavior (e.g., substance abuse, intensified sexual behaviour), impulsivity (e.g., impetuous monetary spending), mood dysregulation, and subsequent interpersonal difficulties [1–3]. While Bipolar type I (BP-I) and Bipolar type II (BD-II) differ mainly in the presence of periods of mania, both can significantly complicate the lives of those diagnosed [1, 2, 4].

BD has a lifetime prevalence of 3.9% within the adult population, with an estimated suicidal risk 20 to 30 times greater than the general population [5, 6]. In fact, among all psychiatric illnesses, research contends that individuals diagnosed with BD actually have the highest suicide risk [e.g., 7, 8], and as such, may be considered a major public health threat requiring advanced assessment into various options for management and treatment.

## BD, social support and bi-directionality

The primary aims of BD treatment are to reduce acute symptomology, improve psychosocial functioning, and diminish the likelihood of relapse/recurrence through pharmacotherapy coupled with psychological interventions [e.g., 9]. Further, the efficacy of psychological therapies may be advanced as contemporary perspectives adopt a more holistic conceptualization of BD impact and management by exploring psychosocial factors such as social support [10–13]. Social support may be generally characterized by feelings of being loved and cared for, relying on others when in need [e.g., 14], and a sense of belonging to groups and/or communities [e.g., 15–17].

Within the context of BD, research tends to report better psychological health for those with more social support [e.g., 18, 19], perhaps by mitigating the severity of the disorder’s symptoms [e.g., 18, 20, 21], helping individuals better cope with life circumstances through the provision of empathy and understanding [e.g., 18], and/or challenging negative rumination thereby averting major mood episodes [e.g., 18]. On the contrary, unhealthy social relationships and interactions may serve to undermine the mental health of those with BD, particularly in situations where social others maintain and/or exhibit a highly stigmatize perspective of BD [e.g., 18], and have little sympathy or understanding of symptoms [e.g., 22]. Consequently, negative social contexts may serve to exacerbate already precarious mental health, potentially acting as a trigger for mania or depression [e.g., 18]. For this reason, it is feasible to explore the distinct impact that negative social interactions (NSIs) might have on wellness and functioning beyond social support factors.

Although investigations consistently indicate that those with BD tend to report less social support compared with non-clinical samples, [e.g., 8, 20], of particular importance are factors that may not only compromise the extent of social networks, but also quality and influence. For instance, studies into SMIs such as BD note the potentially harmful impact of internalized stigma [e.g., 23] which may directly diminish self-esteem and self-efficacy, leading to social withdrawal and a sense of alienation [e.g., 24]. Moreover, comprehensive assessments of the impact of stigma further suggest the potential for a paradoxical response whereby sufferers may be energized by prejudice which may be expressed as righteous anger [23]. This, combined with BD’s defining symptomology such as erratic mood changes, impulsivity [e.g., 25], and engagement in unhealthy coping behaviours [e.g., 26] may collectively serve to affect the extent and quality of social support [e.g., 18, 27]. Such complex social dynamics implicates a bi-directionality which has also been considered in the assessment of social factors in other SMIs such as posttraumatic stress disorder (PTSD), a diagnosis highly comorbid with BD [e.g., 26].

Indeed, social support is one of the most constant and primary covariates in PTSD research [28] with its influence typically interpreted as protective or salutary [e.g., 29], whereby deficiencies may result in poorer mental health such as psychological distress [e.g., 30]. Further, supportive social relations, particularly with family and close friends, have been observed to predict better functioning, and more effective symptom management following a traumatic event [31, 32]. However, since PTSD often presents with sufferers being easily alarmed and irritated, often resorting to problematic drug use, or alcohol consumption [e.g., 33], interpersonal difficulties with family and friends are quite likely [e.g., 34]. Accordingly, research investigating the relationship between social support and PTSD (particularly longitudinal studies) has suggested a causal association that operates the opposite way (i.e., the ‘social erosion hypothesis’) [e.g., 29, 30] whereby irritability, anger, and detachment behaviours serve to erode relationship quality and social support resources/availability [29, 30].

Similar to PTSD/social support investigations, relationship erosion as a consequence of stigma and/or intrusive symptomology is a challenging reality of BD, particularly given that the support of family members and caregivers dedicated to the welfare of sufferers is vital for optimal wellness outcomes [27]. Moreover, research suggests that familial support is especially precarious since those intimately involved also tend to report notable caregiver distress as a function of recurring BD symptoms of loved ones [e.g., 35]. Given the potentially detrimental social costs of BD, it has been proposed that a more complete appreciation of the bi-directional nature of relationship scope and quality, and health status/functioning are essential to both understand and ultimately improve BD interventions involving a psychosocial perspective [e.g., 18].

### BD, resilience, positive mental health and social support

Although adverse mental health consequences of BD have been well documented in the literature, such as higher psychological distress [e.g., 19] and poorer quality of life, [e.g., 25] it has been proposed that SMIs such as BD may also produce circumstances resulting in constructive outcomes [e.g., 22]. For instance, some studies into BD report that hypomanic symptoms (e.g., reduced social inhibitions) may serve to enrich social networks by enabling new connections [18], while a sense of amplified confidence during times of positive affect may facilitate the pursuit of aspiring goals [36]. Moreover, the experience of living with BD could provide opportunities for growth as valued and beneficial psychosocial characteristics may foster a sense of resilience, particularly during negative social circumstances [22]. Indeed, it has been proposed that BD treatment plans may be vastly improved with efforts to explore and enhance the resilience of BD patients [e.g., 37].

Accordingly, studies into BD have explored the concept of resilience as both a dependent variable, as well as an explanatory variable of various mental health outcomes. For example, Choi et al. (2015) explored resilience as a dependent variable in BD patients [37] using the Connor–Davidson Resilience Scale (CD-RISC), an instrument designed to assess four distinct factors including (1) personal competence, (2) confidence and effectiveness, (3) positive coping and secure relationships, and (4) spirituality [38]. Compared with a matched control group, it was observed that BD patients had significantly lower CD-RISC scores, with higher impulsivity and more frequent depressive episodes specifically predictive of lower resilience levels [37].

More recently, a 23-item resilience instrument specific to BD has been developed (i.e., the Resilience Questionnaire for Bipolar Disorder (RBD)) to assess five particular domains including ‘self-management’ (i.e., personal capacity to manage BD), ‘turning point’ (i.e., resolve and commitment to change), ‘self-care’ (i.e., disciplined management one’s own health), ‘self-confidence’ (i.e., self-reliance and self-respectful attitudes and actions), and formal and informal ‘interpersonal support’ (i.e., feeling loved, supported, as well as being informed by others when BD symptoms become active/apparent) [39]. Using the RBD to explore whether, and degree to which domains predicted mental health outcomes of 125 BD patients, it was observed that ‘self-management’, ‘turning point’, ‘self-care’, and ‘self-confidence’ were associated with personal recovery, symptomology, psychosocial functioning, and quality of life measures at baseline, with ‘self-confidence’ predictive of personal recovery at follow-up [1]. Interestingly, it was also observed that the improvement of the ‘self-confidence’ domain mediated the link between ‘interpersonal support’ and ‘self-care’, and subsequent personal recovery at follow-up, suggesting that resilience domains are significantly associated with positive mental health outcomes in BD, with some (including ‘interpersonal support’) predictive of personal recovery at follow-up.

As measures of resilience, both the CD-RISC and RBD feature comparable domains with general themes reflecting a sense of personal competence, self-efficacy, and positive coping as well as distinct social support domains (i.e., ‘secure relationships’ and ‘interpersonal support’ respectively). It is also evident that both instruments reflect the significant conceptual evolution of ‘mental health’ which has advanced beyond a rudimentary notion of the mere presence/absence of mental illness, to involve the degree to which individuals may thrive and adapt in response to various mental health experiences. For example, the World Health Organization’s definition of mental health reflects a state of wellness defined by positive coping with life stressors, efficacious involvement in work and society, and an acute appreciation of one’s potential [40]. As a very comparable construct, positive mental health (PMH) also represents a sense of resilience or flourishing whereby people maintain a sense of control, self-esteem, constructive coping, and self-acceptance [41]. PMH has also been conceived of as a means by which resilient responses are realized [42], as well as a type of defense mechanism as indicated by a person’s perseverance through continued negative occurrences [e.g., 43–45]. In fact, according to Srivastava (2011) in an editorial considering the connection between PMH and resilience, “It will be incomplete to talk about positive mental health without making a mention of resilience” [46].

In terms of specific PMH measures, a 14-item instrument called the Mental Health Continuum – Short Form (MHC-SF) [41] has been used in several national population health surveys (e.g., the CCHS-MH) to capture the degree to which respondents might languish or flourish [e.g., 47]. Similar to resilience measures such as the CD-RISC and RBD, the MHC-SF contains psychological, emotional and social well-being subscales assessing such characteristics as one’s sense of confidence and competence, life satisfaction and wellness, and belongingness to a community (See Methods for more detailed description). Overall, it would appear that conceptualizations of resilience and PMH are quite similar, particularly since they contain distinct social support domains which appear especially prognostic of personal recovery for BD patients [e.g., 1]. Taken together, while establishing the PMH and capacity to flourish may prove valuable to understand degree of resilience in adults with BD, specifically examining whether, and degree to which subtypes of social support, and NSIs predict PMH may further elucidate, and hence help influence one’s propensity toward adaptive responses to facilitate the management and treatment of BD, and self-recovery [e.g., 1].

### Exploring PMH in adults with BD as a function of social support subtypes and NSIs: objectives of the current study

Based upon the preceding, the present study aimed to examine how adults with BD compare with those without in terms of social support, NSIs, and PMH. Additionally, for those with BD, determine whether male and female respondents differ in terms of social support, NSIs, ands PMH, and whether, and extent to which particular social support subtypes, and NSIs predict PMH. Currently, there is limited research assessing the association between psychosocial factors and PMH for individuals diagnosed with BD [10–13], so elucidating the connection may allow for more effective prevention, intervention, and treatment options for those seeking mental health support [48]. Moreover, while research does investigate a probable link between social support and mental health for those with BD, studies directly assessing resilience within the context of social support are scarce [e.g., 18–21].

It is also important to note a literature gap in the potential influence of NSIs on the resilience of those diagnosed with BD. To our knowledge, since BD studies have not considered the NSI/PMH link, the current research aims to advance the groundwork. In line with previous research [e.g., 19], it is hypothesized that levels of social support will positively predict PMH levels in those with BD. Furthermore, while specific literature considering the potential relationship between NSIs and PMH among those with BD does not apparently exist, it is feasible to posit that these variables will be inversely associated.

As a specific note about the BD sample utilized in this research, although studies into BD are widespread, many are likely to have recruited participants from clinical treatment programs whereby patients will have received a substantiated diagnosis by a mental health professional [e.g., 1, 8, 37, 49]. However, the sample featured in the present study involves population health survey respondents which (among many variables) captures adult Canadians reporting BD diagnosed by a health care professional. Since this is a population-based sample, a proportion of respondents, despite reporting the diagnosis, may not have actually received treatment (and hence, excluded from potential sampling frames for BD research), or have opted not to participate in studies promoted through clinical programs. Therefore, by utilizing a large, nationally representative Canadian adult health survey, the current study findings may be more representative of adults managing BD in Canada.

## Method

### Data Collection

This study featured data extracted from the cross-sectional 2012 Canadian Community Health Survey - Mental Health (CCHS-MH) [47] public use data file. Data collection occurred between January 2nd, 2012 and December 31st, 2012, and utilized various methods of participant questioning including in-person, computed-assisted personal interviewing, and over the phone interviews [47].

It is important to note that although more recent CCHS public use micro files exist, the CCHS-MH (2012) data resource was utilized in the current study since it is a specialized mental health version capturing core measures essential to this study, administered to *every* respondent, representing *each* Canadian province. Unfortunately, Statistics Canada has yet to release a more up-to-date version of the CCHS-MH.

### Participants

Survey respondents were Canadians aged 15 years and older randomly sampled from the ten provinces, resulting in a total of 25,113, or a response rate of 68.9% [47]. Individuals living in Aboriginal settlements, the three Canadian territories, and Canadian Forces, as well as those institutionalized were omitted from data collection, and have been assessed to represent less than 3% of the Canadian population [47]. Age categories were arranged in 5-year groupings, ranging between ‘15–19 years’ and ‘80 years or older.’ Adult respondents for the study fell within 20 and 64 years of age, with 563 reporting a lifetime diagnosis of BD.

## Materials

### The social provisions scale 10 items (SPS-10)

Perceived social support was captured by means of a 10-item instrument termed the Social Provisions Scale (SPS-10), with two statements representing five distinct dimensions of social support; i.e., ‘attachment’ (i.e., the perception of emotional closeness with others); ‘guidance’ (i.e., a sense that one feels others are available to provide advice or information); ‘reliable alliance’ (i.e., degree of reliance on others during times of distress); ‘social integration’ (i.e., a sense of belonging to individuals or groups); and ‘reassurance of worth’ (i.e., a belief that one’s competence is acknowledged by others). Respondents rank each statement on a scale between 1 (i.e., ‘strongly agree’) and 4 (i.e., ‘strongly disagree’), and a total SPS-10 score calculated via the summation of responses which range between 0 and a maximum of 40, with a larger values indicating higher levels of overall social support [47, 50]. Administrations of this instrument have revealed excellent internal reliability and construct validity [50].

### The mental health continuum - short form (MHC-SF)

PMH was assessed by means of the MHC-SF [41], a 14-item instrument representing three distinct subscales; i.e., psychological, social, and emotional well-being. In particular, six statements were adapted from Ryff’s psychological well-being model (e.g., ‘confident to think or express your own ideas and opinions’), five from Keyes’ (1998) social well-being model (e.g., ‘that you belonged to a community’), and three from Keyes (2009) dimension of subjective/emotional well-being (i.e., ‘satisfied with life’). Specifically, each question asks, ‘During the past month, how often did you feel…’ with potential responses falling within a six-point scale (i.e., 1 - ‘everyday’ and 6 – ‘never’). Items were subsequently reverse coded, with one point deducted from each score such that a response of ‘never’ resulted in a value of 0, and ‘everyday’ a value of 5. A total MHC-SF score is derived via the summation of question scores, with potential values ranging between 0 and 70 (with higher MHC-SF totals signifying higher/better PMH and a subsequent categorization of ‘flourishing’) [51–53]. Administrations of the MHC-SF have revealed high internal, and moderate test-retest reliability [e.g., 54].

**Negative Social Interactions (NSI) Scale** The potential impact of negative social relationships was captured by means of the Negative Social Interactions (NSI) Scale, a self-report measure assessing whether respondents have ‘regular contact’ with people who ‘are detrimental to … well-being because they are a source of discomfort and stress.’ The scale includes four items prompted by the statement ‘in the past month, how often have others’, followed by (1) ‘made too many demands on you?’, (2) ‘were critical of you and things you did?’, (3) ‘did things that were thoughtless or inconsiderate?’, and (4) ‘acted angry or upset with you?’ with respondents ranking each on a scale of 1 (‘never’) to 4 (‘very often’). Total scores range between 0 and 12, calculated through the summation of these rankings after values are recoded from 1 to 4, to 0–3, with higher numbers indicating more frequent NSIs (Krause, 1995; cited in Statistics Canada, 2013) [47].

### Self-report of bipolar disorder

Among several self-reported chronic conditions, the CCHS-MH (2012) queried the existence of particular mental health conditions ‘diagnosed by a health professional’ that ‘have lasted or were expected to last more than 6 months.’ To specifically assess self-reported bipolar disorder, participants were asked, ‘Do you have a mood disorder such as depression, bipolar disorder, mania or dysthymia?’ with respondents identifying the particular disorder(s) with subsequent agreement [47].

### Assessment of bipolar disorder: lifetime prevalence

After completing the screener question section, respondents proceeded to the CCHS-MH ‘depression and mania modules’ with those responding ‘no’ being exempted from completing items from the disorder module, and not meeting the disorder criteria. The CCHS-MH module questions for BD-I and BD-II disorders were adapted from a World Health Organization version of the Composite International Diagnostic Interview (WHO-CIDI) [47], a standardized protocol for the valuation of mental disorders and conditions predicated on definitions and criteria of the Diagnostic and Statistical Manual of Mental Disorders [55] as well as the International Classification of Diseases and Related Health Problems [56]. Computer-based algorithms derived the lifetime criteria for each disorder as a function of participant responses, along with a requirement that comorbid occupational and social functioning impairments were also evident.

### CIDI classification of BD-I, BD-II and Omnibus BD

In the CCHS-MH (2012), three types of BD types are derived, BD-I, BD-II, and omnibus BD. The criteria for a BD-I or BD-II disorder diagnosis were predicated on McDonald et al. (2015) whereby a BD-I classification was assigned to respondents who experienced six or more symptoms of mania, and two or more ‘super-symptoms’ which involved being ‘exceedingly friendly,’ ‘acting erroneously,’ ‘getting involved with things that lack good judgement,’ ‘managing money poorly,’ or ‘thinking they are a different person or connected to a famous person.’ [47] A BD-II classification was assigned to respondents who experienced an ‘elevated mood lasting a week or longer,’ three or more mania symptoms, ‘euphoria,’ or ‘racing thoughts,’ and ‘marked impairment in social or occupational functioning’, as well as one or more episodes of major depression during their lifetime, with no criteria indicating a manic episode during their lifetime [47]. The omnibus BD measure involves individuals who satisfy the criteria for BD-I disorder or hypomania episode, that includes BD-II disorder. It is defined by 7 or more days (less if one is hospitalized) of heightened or agitated mood plus a particular number and grouping of other manic symptoms including racing thoughts, excessive talking, overspending, diminished desire for sleep, more frequent pleasure seeking behaviour, or inflated self-confidence. Many individuals also experience one or more depressive episodes [40]. Moreover, since BD-II is a subset of hypomania, all those with lifetime BD-II satisfy the criteria for hypomania. Therefore, lifetime BD might be simply derived as the summation of those with BD-I and hypomania. The CCHS-MH includes the BD-II to enable a more detailed appreciation of the population incorporated in the BD variable [47].

### Sociodemographic and socioeconomic variables

Sociodemographic variables were categorical and featured gender (i.e., male, female), age (i.e., 20–34, 35–39, 40–44, 45–49, 50–54, 55–59, 60–64), and marital status (i.e., married, common law, widowed, divorced or separated, and single). The socioeconomic variable of interest was personal income presented in Canadian dollars (i.e., less than $10,000, $10,000-$19,999, $20,000-$29,999, $30,000-$39,999, $40,000-$49,999, and $50,000 and above) [47].

### Statistical analysis

Data from the CCHS-MH were analyzed using version 27.0 of the Statistical Package for the Social Sciences (SPSS). Preliminary statistical analyses involved comparisons between adult respondents with BD and an age, gender, and frequency-matched, non-BD sample created by means of random sampling non-BD respondents from the CCHS-MH to equal the number of BD respondents that fell within each age by gender cell. Initially, Chi square tests were conducted to determine whether marital status and personal income categories were dependent on a reported BD diagnosis. Secondly, independent sample *t* tests were carried out to compare SPS-10 and subtype means between those reporting a BD diagnosis and the matched non-BD sample, while independent sample *t* tests compared MHC-SF scores (measuring PMH) and NSI score means between those reporting a BD diagnosis and the matched non-BD sample. Thirdly, independent sample *t* tests were conducted to compare male and female respondents with a BD diagnosis in terms of SPS-10 (i.e., overall and for each subtype), as well as MHC-SF and NSI score means. Lastly, hierarchical regressions were performed separately for male and female respondents with a BD diagnosis to explore whether SPS-10 subtypes (assessed in block 2) and NSI (assessed in block 3) predicted PMH beyond variance accounted for by respondent age and income in block 1.

## Results

The CCHS-MH data file contains a total of 16,972 respondents between 20 and 64 years of age, with 563 (i.e., 282 males and 281 females) reporting lifetime BD, a prevalence of 3.3%. Within the BD sample, 91 males and 98 females were categorized BD-1, with 191 males and 183 females identified as omnibus BD, of which 62 males and 66 females were categorized as BD-II.

Table 1 provides the age and gender breakdown of the BD sample, and a matched, non-BD sample randomly generated based on the BD sample’s age, gender, and frequency characteristics. Table 1 also reveals that marital status was significantly dependent a BD diagnosis, with a similar trend exhibited by males and females (*χ*² = 30.9, *p* < .001 and*χ*² = 34.6, *p* < .001 respectively). For instance, compared to the non-BD matched sample, males and females reporting BD were less likely to be married (i.e., 21.4 vs. 41.5% and 26.6 vs. 45.9% respectively), more likely to be single (i.e., 51.2 vs. 34.0% and 34.5 vs. 24.4% respectively), and more likely to be separated or divorced, a trend particularly evident for females (i.e., 17.1 vs. 12.1% and 23.7 vs. 11.1% respectively).

**Table 1:** Age Group, Marital Status, and Personal Income Frequencies and Percentages by Gender for Respondents with BD, and an Age, Gender and Frequency Matched Non-BD Sample

		Percent (N)							
		Male				Female			
		BD	Non-BD	χ2	*p*	BD	Non-BD	χ2	*p*
		(N = 282)	(N = 282)			(N = 281)	(N = 281)		
Age									
20 to 24 years		16.3(46)	16.3(46)	0.00	1.00	15.7(44)	15.7(44)	0.00	1.00
25 to 29 years		9.2(26)	9.2(26)			9.3(26)	9.3(26)		
30 to 34 years		12.0(34)	12.0(34)			11.0(31)	11.0(31)		
35 to 39 years		14.5(41)	14.5(41)			7.8(22)	7.8(22)		
40 to 44 years		14.5(41)	14.5(41)			10.3(29)	10.3(29)		
45 to 49 years		11.0(31)	11.0(31)			12.1(34)	12.1(34)		
50 to 54 years		7.4(21)	7.4(21)			12.5(35)	12.5(35)		
55 to 59 years		11.3(32)	11.3(32)			11.7(33)	11.7(33)		
60 to 64 years		3.5(10)	3.5(10)			9.6(27)	9.6(27)		
Marital Status									
	Married	21.4(60)	41.5(117)	30.9	0.00	26.6(74)	45.9(128)	34.6	0.00
	Common-Law	10.0(28)	12.1(34)			11.5(32)	16.1(45)		
	Widowed	0.4(1)	0.4(1)			3.6(10)	2.5(7)		
	Divorced/Separated	17.1(48)	12.1(34)			23.7(66)	11.1(31)		
	Single	51.2(144)	34.0(96)			34.5(96)	24.4(68)		
Personal Income									
	< $10,000	3.3(9)	3.5(9)	41.6	0.00	10.2(27)	10.4(27)	32.0	0.00
	$10,000-$19,999	20.0(54)	7.7(20)			30.8(82)	15.1(39)		
	$20,000-$29,999	23.7(64)	19.2(50)			29.3(78)	24.3(63)		
	$30,000-$39,999	15.9(43)	8.5(22)			12.0(32)	15.1(39)		
	$40,000-$49,999	11.5(31)	11.9(31)			5.3(14)	8.5(22)		
	$50,000 +	25.6(69)	49.2(128)			12.4(33)	26.6(69)		

Table 1 also reveals a significant Chi Square for personal income indicating that such was dependent on BD diagnosis, a trend evident for both males and female respondents (*χ*² = 41.6, *p* < .001 and*χ*² = 32.0, *p* < .001 respectively). For instance, compared to the non-BD matched sample, males and females with BD were more likely to report an income under $30,000 (i.e., 47.0 vs. 30.4% and 70.3 vs. 49.8% respectively), and less likely to report an income $50,000 or more (i.e., 25.6 vs. 34.0% and 12.4 vs. 26.6% respectively).

### Comparison of adults with BD to the matched non-BD adult sample

An independent sample *t* test assessing the difference in overall SPS-10 score between the BD sample (*n* = 552, *M* = 33.37, *SD* = 5.85) and the matched, non-BD sample (*n* = 554, *M* = 36.01, *SD* = 4.34) revealed that the BD sample was significantly lower, *t*(1104) = -8.54, *p* < .001, 95% CI [-3.33, -2.04] with a medium effect size, *d* = − 0.514 (See Table 2). Independent sample *t* tests comparing each SPS-10 subscale revealed significantly lower scores for the BD respondents compared with the matched non-BD sample, i.e.; ‘attachment’ (*n* = 559, *M* = 6.77, *SD* = 1.33 vs. *n* = 562, *M* = 7.26, *SD* = 0.99), *t*(1119) = -7.04, *p* < .001, *d* = − 0.421, 95% CI [-0.63, − 0.36]; ‘guidance’ (*n* = 561, *M* = 6.84, *SD* = 1.38 vs. *n* = 562, *M* = 7.36, *SD* = 1.01), *t*(1121) = -7.17, *p* < .001, 95% CI [-0.66, − 0.38]; ‘reliable alliance’ (*n* = 561, *M* = 6.90, *SD* = 1.27 vs. *n* = 562, *M* = 7.34, *SD* = 0.91), *t*(1121) = -6.704, *p* < .001, 95% CI [-0.57, − 0.31]; ‘social integration’ (*n* = 557, *M* = 6.32, *SD* = 1.42 vs. *n* = 559, *M* = 6.98, *SD* = 1.10), *t*(1114) = -8.687, *p* < .001, 95% CI [-0.81, − 0.51]; and ‘reassurance of worth’ (*n* = 556, *M* = 6.52, *SD* = 1.33 vs. *n* = 557, *M* = 7.03, *SD* = 1.04), *t*(1111) = -7.201, *p* < .001, 95% CI [-0.66, − 0.38] with medium effect sizes ranging from *d* = − 0.400 for ‘reliable alliance’ to *d* = − 0.520 for ‘social integration’.

**Table 2:** Overall Social Provision Scale (SPS) and Subtype, Positive Mental Health (PMH), and Negative Social Interactions (NSIs) Means and Standard Deviations for BD Sample and Matched Non-BD Sample

Variable	BD Sample (*n* = 563)		Non-BD Sample (*n =* 563*)*		Sig
	*M*	*SD*	*M*	*SD*	
Overall Social Provision Scale	33.37	5.85	36.01	4.34	***
Attachment	6.77	1.33	7.26	0.99	***
Guidance	6.84	1.38	7.36	1.01	***
Reliable Alliance	6.90	1.27	7.34	0.91	***
Social Integration	6.32	1.42	6.98	1.10	***
Reassurance of Worth	6.52	1.33	7.03	1.04	***
Positive Mental Health	42.38	14.81	53.0	11.42	***
Negative Social Interactions	5.01	3.02	3.13	2.43	***

Note. ***p < .001

Subsequent independent sample *t*-tests revealed significantly lower PMH scores for BD respondents (*n* = 531, *M* = 42.38, *SD* = 14.81) compared with the non-BD matched sample (*n* = 533, *M* = 53.0, *SD* = 11.42), *t*(1062) = -13.081, p < .001, 95% CI [-12.20, -9.01] with a large effect size (i.e., *d* = − 0.802), as well as significantly higher NSI scores (*n* = 556, *M* = 5.01, *SD* = 3.02 vs. *n* = 556, *M* = 3.01, *SD* = 2.44), *t*(1110) = 11.401, p < .001,, 95% CI [1.55, 2.20], also with a large effect size (i.e., *d* = 0.684).

### Comparison of SPS-10, PMH, and NSIs between males and females with BD

Table 3 presents means and standard deviations for overall SPS-10 and subtype, PMH and NSI for male and female respondents with a BD diagnosis. A significant difference was found in overall SPS-10 scores between males with BD (*n* = 276, *M* = 32.85, *SD* = 5.97) and females with BD (*n* = 276, *M* = 33.88, *SD* = 5.68), with males having lower scores, *t*(550) = -2.074, *p* < .05, 95% CI [-2.004, − 0.054] (and a small effect size *d* = 0.177). As for each SPS-10 subtype, males with BD had significantly lower scores in terms of ‘attachment’ (*n* = 280, *M* = 5.58, *SD* = 1.39 vs. *n* = 279, *M* = 6.96, *SD* = 1.24), *t*(557) = -3.456, *p* < .05, 95% CI [-0.605, − 0.166]; and ‘guidance’ (*n* = 281, *M* = 6.69, *SD* = 1.43 vs. *n* = 280, *M* = 6.99, *SD* = 1.32), *t*(559) = -2.510, *p* < .05, 95% CI [-0.520, − 0.063], both with small effect sizes (i.e., *d* = 0.292 and *d* = 0.212 respectively). An independent sample *t* test also revealed a significant difference in NSI scores between males with BD (*n* = 277, *M* = 4.71, *SD* = 2.89) and females with BD (*n* = 279, *M* = 5.30, *SD* = 3.11), with females having higher scores, *t*(554) = -2.330, *p* < .05, 95% CI [-1.094, − 0.094], also with a small effect size (i.e., *d* = 0.198).

**Table 3:** Overall Social Provision Scale (SPS) and subtype, Positive Mental Health (PMH) and Negative Social Interactions (NSI) Means and Standard Deviations for the Male and Female BD Sample

Variable	Females with BD (n = 281)		Males with BD (n = 282)		
	*M*	*SD*	*M*	*SD*	Sig
Overall Social Provision Scale	33.88	5.68	32.85	5.97	*
Attachment	6.96	1.24	6.58	1.39	*
Guidance	6.99	1.32	6.69	1.43	*
Reliable Alliance	7.00	1.26	6.80	1.28	*ns*
Social Integration	6.35	1.45	6.29	1.39	*ns*
Reassurance of Worth	6.56	1.35	6.48	1.32	*ns*
Positive Mental Health	42.14	14.42	42.62	15.21	*ns*
Negative Social Interactions	5.30	3.11	4.71	2.89	*

Note. * p < .05

### Predicting PMH with SPS-10 subtypes and NSIs for BD males and females separately

A hierarchical regression was conducted to explore whether SPS-10 subtype scores (entered in block 2) and NSI scores (entered in block 3) predicted PMH for male respondents with BD, after controlling for age and income in block 1. Accordingly, it was observed that income positively predicted PMH in block 1, while age was inversely associated, accounting for 11.9% of the variance (F(2,249) = 17.74, *p* < .001. The results further revealed that two SPS-10 subscales (i.e., ‘social integration’, and ‘reassurance of worth’) significantly and positively predicted PMH in block 2, accounting for an additional 36.9% of the variance (F(7,249) = 34.95, *p* < .001), while NSI scores significantly and inversely predicted PMH, accounting for a further 2.8% (F(8,249) = 34.14, *p* < .001) in block 3 (See Table 4 for statistical results).

**Table 4:** Summary of Hierarchical Regression Analysis for Variables Predicting Positive Mental Health for Males with BD

Variable	Block 1			Block 2			Block 3		
	*B*	*SE*	*β*	*B*	*SE*	*β*	*B*	*SE*	*β*
Age	-1.277	0.367	− 0.207*	− 0.455	0.287	− 0.074	− 0.523	0.279	− 0.085
Income	2.720	0.565	0.286**	1.143	0.448	0.120*	1.199	0.436	0.126*
Attachment				1.784	0.938	0.163	1.977	0.914	0.181*
Guidance				0.193	0.978	0.018	0.299	0.952	0.028
Reliable alliance				0.408	0.898	0.036	− 0.187	0.888	− 0.016
Social Integration				3.375	0.784	0.312**	2.950	0.771	0.273**
Reassurance of worth				2.365	0.797	0.206*	2.438	0.776	0.213*
Negative Social Interactions							− 0.944	0.247	− 0.178**
*R* ^*2*^			0.119			0.488			0.516
* F* for change in *R*^*2*^			17.74**			34.95**			34.14**

Note. * p < .05

** p < .001

For the female sample diagnosed with BD, a hierarchical regression was conducted to explore whether SPS-10 subtype scores (entered in block 2) and NSI scores (entered in block 3) predicted PMH for female BD respondents, after controlling for age and income in block 1. The results indicated that income was positively associated with PMH, accounting for 5.3% of the variance (F(2,244) = 7.85, *p* < .001). An assessment of SPS-10 subtypes in block 2 revealed that two (i.e., ‘social integration’ and ‘reassurance of worth’) significantly and positively predicted PMH accounting for an additional 23.4% of the variance (F(7,249) = 15.06, *p* < .001), while NSI significantly and inversely predicted PMH in block 3 accounting for a further 2.9% of the variance (F(8,244) = 12.11, *p* < .001) (See Table 5 for statistical results).

**Table 5:** Summary of Hierarchical Regression Analysis for Variables Predicting Positive Mental Health for Females with BD

Variable	Block 1			Block 2			Block 3		
	*B*	*SE*	*β*	*B*	*SE*	*β*	*B*	*SE*	*β*
Age	− 0.158	0.344	− 0.029	0.079	0.300	0.014	− 0.074	0.298	− 0.014
Income	2.430	0.614	0.248**	1.037	0.560	0.106	1.025	0.549	0.105
Attachment				− 0.947	1.136	− 0.082	− 0.486	1.122	− 0.042
Guidance				1.536	1.129	0.133	1.228	1.110	0.107
Reliable alliance				-1.035	1.026	− 0.087	-1.264	1.007	− 0.107
Social Integration				3.693	0.864	0.379**	3.297	0.854	0.338**
Reassurance of worth				2.119	0.918	0.196*	1.927	0.901	0.178*
Negative Social Interactions							− 0.870	0.262	− 0.188*
*R* ^*2*^			0.053			0.287			0.316
* F* for change in *R*^*2*^			7.85**			15.06**			12.11**

Note. * p < .05

** p < .001

## Discussion

Comparable to other rates reported in the literature [e.g., 5, 6], it was observed that 3.3% of Canadians between 20 and 64 years of age in the CCHS-MH were assessed as having a lifetime prevalence of BD. In terms of social functioning, research indicates that individuals with BD tend to have difficulties navigating and maintaining social relationships [e.g., 57, 58], and not surprisingly, our results are consistent in this regard [e.g., 19]. Specifically, respondents with BD reported less overall social support, and for each subtype (i.e., ‘attachment’, ‘guidance’, ‘reliable alliance’, ‘social integration’, and ‘reassurance of worth’) compared with that reported by non-BD matched sample. Moreover, an assessment of marital status revealed that respondents with BD were also more likely to report being single, separated or divorced.

Our study also examined potential gender differences among adults diagnosed with BD, and specifically observed that female respondents reported significantly higher overall social support, as well as higher ‘attachment’ (i.e., feeling an emotional connection with someone) and ‘guidance’ (i.e., feeling like they have someone to pose questions to, and receive advice from). This finding was expected, as similar research revealed comparable, yet inverse results when predicting psychological distress [i.e., 58]. Additionally, within the context of depression, other studies have observed that females tend to seek out social support more readily, as well as use their social support networks to gain self-awareness of their challenges and issues more than men [e.g., 59].

As expected, compared to the matched non-BD sample, levels of PMH were significantly lower for respondents with BD, intuitive findings considering likely quality of life impairments associated with the disorder, as well as substantial suicide risk [e.g., 1, 4, 7]. Interestingly however, gender differences were *not* observed within the BD sample with respect to PMH levels, a finding certainly worthy of further investigation, particularly given that other studies into similarly distinctive forms of SMIs (i.e., schizophrenia) have observed that females tend to have superior PMH when compared to males with the same diagnosis [e.g., 60].

In terms of NSIs, respondents with BD reported significantly higher levels than the matched non-BD sample, and while research in this area is sparse, a few explanations seem plausible. Firstly, it is feasible that heighted public stigma may play a role [e.g., 24], particularly given evidence to suggest that BD tends to be appraised more negatively than conditions such as depression [e.g., 61]. Research also suggests that those with BD are more likely to be perceived as blameworthy for their illness [e.g., 62], judgmental beliefs that may facilitate deleterious emotional interactions [63]. Moreover, the resulting internalized stigma associated with BD may compromise self-esteem and self-efficacy [e.g., 23], facilitating social withdrawal [e.g., 24], and/or paradoxically, the expression of righteous anger brought about by the experience of prejudice [23].

In addition to the impact of stigma, higher NSI scores might also be a function of characteristic BD symptomology such as risk-taking, impulsivity, and/or mood dysregulation that could cause turmoil in relationships [e.g., 2]. As previous considered, social erosion and compromised relationship quality seem quite likely when considering the bi-directionality of social support within the context of BD [e.g., 18, 27]. Moreover, whether higher NSI scale scores are a consequence of a response to public stigma on the part of those with BD, reactivity to BD symptomology by social others, preconceived notions of social toxicity believed to characterize the disorder, a sense of burnout or frustration being expressed by intimate partners or close family members after supporting loved ones through cycles of the disorder, or perhaps conflict surfacing within the workplace, negative relationships appear to exert distinctive influence beyond social support factors, and may significantly interfere with the likelihood of resilience or flourishing, and as such, are certainly worthy of more continued assessment.

In terms of NSIs and gender differences, females with BD reported higher scores, an intriguing observation given that they also reported higher levels of overall social support. While further research is warranted to explore the intricacies of this observation, particularly within the context of BD, perhaps this finding may be considered in light of studies that have explored gender differences in rumination (i.e., repetitive negative thought processes) which have been conducted to assess plausible reasons why women have notably higher prevalence rates of depression [e.g., 64]. Indeed, some research suggests that those with a ruminative coping style tend to pursue social support more frequently, and since such a coping style is more likely in females, perhaps social support may function as a means to fixate on challenging situations and/or distorted perceptions [e.g., 65], and this might predict a higher risk for NSIs. In all, perhaps, the social support/NSIs link may represent, and be characterized by a specific type of invasive cogitation; i.e., ‘social rumination’ which may be predicated primarily on gender.

Our hypothesis that social support would positively predict PMH for adults with BD was supported, with two particular dimensions, namely ‘reassurance of worth’ (i.e., a sense that one’s abilities are recognized by others) and ‘social integration’ (i.e., a person’s sense of belonging to a group) emerging as significant factors for males and females. The plausible salutary influence of these particular social support subtypes are implicated in findings reported elsewhere in the literature, but with a significant negative association with psychological distress as the dependent measure [i.e., 19]. Such findings also seem reflected in qualitative studies into BD and social support. For instance, in Owen et al. (2017)’s investigation into the reciprocal association between BD and social relationships, semi-structured interviews with individuals with BD revealed that when respondents felt their disorder was defined by others as a weakness (perhaps indicating a *lack* of reassurance of worth), such served to increase the likelihood of social problems, and subsequent negative mental health outcomes [18]. Also congruent with ‘reassurance of worth’ dimension, Owen et al. (2017) revealed that when BD respondents reported others’ sympathy and understanding, effective personal coping was more likely [18]. Similarly, Galvez et al. (2011) proposed that when others are interested in, and sympathetic to BD experiences, such reassurance may positively modify negative cognitions [22]. Additionally, it may be reasoned that more perceived social acceptance (as indicated in higher ‘social integration’, or sense of belonging) may result in lower perceived stigma, and hence higher PMH.

In all, within the context of social support, perhaps *how* people make us feel is key in fostering of a sense of PMH. That is, higher levels of ‘reassurance of worth’ directly affirm one’s value, and perhaps counteract the impact of stigma. Similarly, feeling a sense of belongingness and acceptance as indicated in higher levels of ‘social integration’ may serve to enhance feelings of self-worth, and also reduce stigma. While these findings make intuitive sense regardless of affliction, it appears particularly important for those living with BD, who have likely paid a social price due to the realities of the illness. Indeed, social erosion is a probable outcome predicated on the expense loved ones have paid in attempting to care for and support people in the throes of their illness. It would also appear that NSIs may play a unique role in the degree to which BD sufferers experience PMH. Previous research has reported that NSIs tend to predict low interpersonal trust, external control beliefs, and the exhibition of dysfunctional attitudes, factors predictive of psychological distress via self-esteem [i.e., 66].

An examination of NSI scores revealed an inverse relationship with PMH for both male and females with BD, findings comparable to those reported in other research [e.g., 67]. Similarly, NSIs have been observed to be predictive of poorer mental health, and potential triggers for mania or depression episodes [e.g., 18]. These results highlight the importance of supporting those with BD in terms of methods to cope with, and manage NSIs they might experience in daily life. Future research could further assess the types and nuances of NSIs that tend to compromise PMH, as well as whether and how particular sources might play a role (i.e., family members, intimate partners, close friends, acquaintances). Subsequent studies might also consider NSI reduction through interventions designed to diminish public stigma toward BD, as well as improving the coping skills of family or other social supports who are primary caregivers to those with BD.

As a final suggestion for future research, the intuitive interpretation of the social support/PMH link is that social support operates in a salutary manner to enhance an individual’s sense of mental health, wellness and resilience. However, it is important to note that findings from Echezarraga et al. (2018) provide evidence to suggest that the association may also work the other way whereby higher PMH enhances one’s capacity to seek out, and more constructively engage in (and benefit from) social relationships [1]. Specifically, that study’s longitudinal design allowed for the observation that an improvement in self-confidence served to mediate an association between interpersonal support and self-care, and subsequent personal recovery at follow-up. Perhaps such findings implicate a social link between resilience domains and positive mental health in those with BD, and hence a greater likelihood for personal recovery. Indeed, future BD studies could measure RBD domains, and social support subscales at baseline to parse out whether subsequent changes in the various sub measures predict recovery at follow up.

### Limitations

There are several limitations within this study that should be noted. The first relates to the exclusion criteria of respondents when the CCHS-MH data were originally collected whereby those institutionalized, residing on indigenous settlements, and Canadian forces members were not included in the sampling frame. While estimated to represent 3% of the population, generalizability to these specific groups may be precarious. Secondly, it is very important to emphasize that this is a correlational study and thus causation cannot be inferred. Hence, any language that seems to insinuate causality is completely unintentional. Thirdly, data associated with medication use and/or other treatments for mental health management were not captured nor controlled in this study, and we acknowledge that such are likely factors that could have influenced levels of PMH. Lastly, given the self-report nature of population health surveys, while CCHS-MH modules on BD-I and BD-II disorders were based on a recognized World Health Organization version of the Composite International Diagnostic Interview, an individual’s diagnosis of BD may not have been verified by an appropriate medical professional.

## Conclusions

Overall, Canadians adults with BD report less social support overall, and across each subtype, experience lower levels of PMH, and higher NSIs compared to adults without the diagnosis. In terms of gender, males with BD appear to report less overall social support, specifically in terms of ‘attachment’ and ‘guidance’, while females with BD tend to report higher NSIs. It was also revealed that ‘social integration’ and ‘reassurance of worth’ positively predicted PMH for both males and females with BD, with NSIs also accounting for unique variance beyond social support, predicting lower levels of PMH. Such observations can aid in the development of treatment programs and therapies for individuals suffering from BD. It would seem that social support (or lack thereof) is important to consider in promoting resilience, and given the gender lens that was applied in this study, perhaps males may see greater success in treatment when focusing on guidance and attachment-based social support (i.e., receiving advice and feeling an emotional connection with others), while females may benefit from tailored treatment surrounding how to cope with NSIs.

## Data Availability

The datasets used and/or analyzed during the current study available from the corresponding author on reasonable request.

## Declarations

Experimental protocols were approved by the *Health Canada and Public Health Agency of Canada (PHAC) Research Ethics Board (REB)*, and.
